# Synthesis of PVA-Based Hydrogels for Biomedical Applications: Recent Trends and Advances

**DOI:** 10.3390/gels11020088

**Published:** 2025-01-23

**Authors:** Mohammad Mizanur Rahman Khan, Md. Mahamudul Hasan Rumon

**Affiliations:** 1 Department of Mechanical Engineering, Gachon University, 1342 Seongnam-daero, Sujeong-gu, Seongnam-si 13120, Gyeonggi-do, Republic of Korea; 2 Department of Chemistry, Indiana University of Bloomington, Bloomington, IN 47405, USA; mdhasa@iu.edu

**Keywords:** PVA-based hydrogels, synthesis, tissue engineering, drug delivery, wound dressing, contact lenses, biomedical applications

## Abstract

There is ongoing research for biomedical applications of polyvinyl alcohol (PVA)-based hydrogels; however, the execution of this has not yet been achieved at an appropriate level for commercialization. Advanced perception is necessary for the design and synthesis of suitable materials, such as PVA-based hydrogel for biomedical applications. Among polymers, PVA-based hydrogel has drawn great interest in biomedical applications owing to their attractive potential with characteristics such as good biocompatibility, great mechanical strength, and apposite water content. By designing the suitable synthesis approach and investigating the hydrogel structure, PVA-based hydrogels can attain superb cytocompatibility, flexibility, and antimicrobial activities, signifying that it is a good candidate for tissue engineering and regenerative medicine, drug delivery, wound dressing, contact lenses, and other fields. In this review, we highlight the current progresses on the synthesis of PVA-based hydrogels for biomedical applications explaining their diverse usage across a variety of areas. We explain numerous synthesis techniques and related phenomena for biomedical applications based on these materials. This review may stipulate a wide reference for future acumens of PVA-based hydrogel materials for their extensive applications in biomedical fields.

## 1. Introduction

PVA-based hydrogels possess excellent mechanical properties with superior biocompatibility, which makes them suitable for biomedical applications [1,2,3]. There are numerous methods to synthesize such hydrogels, including cryogelation, which is the repeated cycling of freezing/thawing in aqueous solutions; the non-cryogenic physical gelation of PVA, the chemical crosslinking of PVA, etc. The synthesis of PVA-based hydrogels through these techniques produces materials with superior mechanical characteristics, excellent biocompatibility, and good chemical stability [2,4]. In this context, the synthesis of PVA-based hydrogels with a controllable molecular weight and a suitable supramolecular connection of polymers may help with their application within the biomedical sectors. Therefore, among numerous materials used in the preparation of hydrogels, PVA is of great interest to the scientific community, owing to its distinctive features, such as water solubility, biodegradability, responsiveness to external stimuli, and the ability to form steady networks through diverse cross-linking approaches [5,6]. These potential characteristics of PVA-based hydrogels make their position as a significant polymeric material in the dominion of hydrogel design and research [7,8,9,10,11]. Thus, PVA-based hydrogels can be considered as ideal materials for diverse applications, such as environmental remediation, biomedicine, and cutting-edge technology improvement.

PVA-based hydrogels are usually synthesized through a number of approaches, including physical and chemical cross-linking techniques [12,13,14,15]. Typical methods, like repeated freezing/thawing cycles and non-cryogenic physical gelation of PVA, were employed for the synthesis of PVA-based hydrogels [16,17]. These techniques can modify the numerous properties of PVA hydrogels, focusing on challenges including mechanical limitations and poor functionality [18,19]. Thus, it is crucial to design and synthesize PVA-based hydrogels which can overcome these limitations for their successful application in biomedical fields [20,21].

Therefore, researchers are focusing on improving the enactment of PVA-based hydrogels utilizing various synthesis strategies and generating PVA-based composite materials, incorporating nanostructured materials, and forming multi-network systems [22,23]. These kinds of trials have occurred with notable electrical conductivity enrichments, mechanical characters, self-healing competences, and sensitivity to environmental stimuli [24,25,26]. Accordingly, the versatility of the applications of PVA-based hydrogels includes drug delivery, regenerative medicine, wound dressing, contact lenses, water purification, and cartilage substitution in joint and meniscal applications [24,27]. Furthermore, these hydrogel materials have wide applications in civil engineering as well as in energy storage, flexible devices, and smart actuators [15,22,23,28].

While several recent reviews have examined PVA-based hydrogels, this manuscript offers a distinctive perspective by exploring the direct connections between the chemical properties of PVA hydrogels and their corresponding biological functionalities. Unlike previous works, we provide a structured evaluation tailored to application-specific requirements—such as biocompatibility, mechanical strength, and biodegradability—emphasizing strategies for modifying and optimizing these properties to meet the unique demands of key biomedical applications, including wound treatment, drug delivery, and tissue engineering. Furthermore, we integrate emerging insights, practical examples, and innovative approaches to fill gaps in the current literature, presenting a focused, application-oriented analysis. These include the incorporation of nanostructured materials, the creation of multi-network systems, and the use of advanced cross-linking techniques to improve electrical conductivity, mechanical strength, self-healing capabilities, and environmental responsiveness. Through these advancements, versatile PVA-based hydrogels can be designed for a broad range of applications in drug delivery, regenerative medicine, wound dressing, water purification, energy storage, and smart actuators. By addressing critical challenges in PVA hydrogel design, this work aims to enhance their utility in the biomedical and technological fields. We discuss the latest progress in the design and fabrication of PVA-based hydrogels, highlighting their potential for numerous biomedical applications. Ultimately, this review aims to provide a comprehensive understanding of the current state of the field and to inspire future innovations in PVA-based hydrogel research.

## 2. Synthesis Techniques of PVA Hydrogels

Polyvinyl alcohol (PVA) hydrogels are engineered using a variety of innovative methods to interlink PVA chains into a three-dimensional (3D) porous network [29,30,31]. This network is the foundation for hydrogels with a wide range of tunable properties from soft, flexible structures to more rigid and robust forms, all tailored for specific applications [32,33]. These techniques involve a range of interactions, from the weaker forces of physical cross-linking, such as hydrogen bonds, to the more permanent and stronger covalent bonds [34,35,36]. As a result, hydrogels with customized characteristics, such as stiffness, porosity, and water retention, can be precisely synthesized [37,38,39]. Methods include physical approaches (such as freezing/thawing cycles, directional freezing, UV or gamma irradiation, and heat treatments) [17,40], chemical processes (using cross-linking agents, copolymerization, or modifications of the hydroxyl (OH) groups), or a combination of both [32,41]. Various synthesis techniques for PVA hydrogels, with their advantages and disadvantages, are represented in Table 1.

Among these, PVA hydrogels formed through physical cross-linking are particularly valued owing to their outstanding biocompatibility, non-toxicity, and high capacity for absorbing water [42]. These hydrogels also possess tunable viscoelastic properties, making them highly adaptable for use in the biomedical and pharmaceutical fields. Physical cross-linking typically occurs through non-covalent interactions, such as hydrogen bonding, Van der Waals forces, and ionic interactions, which do not alter the PVA backbone chemically—an attractive feature for environmentally friendly applications [43]. When stronger covalent cross-links are introduced, typically using cross-linking agents, PVA hydrogels demonstrate improved mechanical strength, thermal stability, and resistance to solvents [44]. However, this increased strength often comes at the expense of biodegradability, with the potential for undesirable by-products, which can limit the use of chemically cross-linked hydrogels in some applications, particularly in biomaterials and drug delivery systems [45,46]. In response, non-covalent bonding approaches are often preferred. Researchers are increasingly turning to natural cross-linking agents—such as genipin and tannic acid—to reduce the risks associated with chemical cross-linking while still achieving desirable properties. Despite these challenges, chemical cross-linking is essential in applications where high mechanical strength and long-term stability are required, such as membrane production, filtration, and tissue engineering [44].

The balance between mechanical strength and swelling capacity is a defining feature of hydrogels [47,48]. These properties are often inversely related—stronger hydrogels, with higher cross-linking density, tend to absorb less water, while more swollen hydrogels are softer and less rigid. This interplay between strength and water retention makes hydrogels versatile materials for applications such as wound healing, drug delivery, and tissue regeneration, where the optimal balance of water absorption and mechanical properties is essential [49,50,51,52,53]. Researchers are continuously improving fabrication methods to create PVA-based hydrogels that offer multifunctional properties with minimal cost and effort.

Despite significant progress in PVA-based hydrogel research, several key limitations continue to restrict their broader adoption in healthcare [5]. First, achieving the precise mechanical and structural properties required for diverse clinical scenarios can be challenging, as even minor changes in manufacturing conditions can affect gel uniformity and performance [54]. Second, scalability remains a concern: producing consistent, high-quality hydrogels on an industrial scale can be complex and costly, especially when advanced cross-linking methods or nanomaterial integrations are involved [55]. Third, while PVA itself is generally considered safe, the overall biocompatibility of composite or multi-network hydrogels depends on additional components and processing steps, which must be thoroughly evaluated and approved by regulatory bodies [56]. Finally, long-term stability and degradation behavior under physiological conditions need more rigorous study, as these factors influence both safety and effectiveness in medical applications [57]. Addressing these challenges through improved synthesis methods, extensive biocompatibility testing, and standardized manufacturing protocols, PVA-based hydrogels may offer more practical applications in the biomedical sector.

**Table 1:** Various synthesis techniques for PVA hydrogels with their advantages and disadvantages are represented.

Hydrogel Ingredients	Fabrication Method	Results	Limitation	Application	Ref.
SA, and PVA	Freeze–thaw cycle	Enhanced mechanical performances with good biocompatibility and most importantly, controlled drug release depending on pH	Reduced mechanical stability specially at pH 8.0	Drug delivery	[58]
PAA, modified PVA, and β-CD	Free radical	Controlled swelling and drug release which depend on pH without compromising the antioxidant and antibacterial properties	Long-term stability not evaluated	Drug delivery	[59]
Modified CS/PVA	Cross-linked with borax and OSA.	Enhanced injectability and self-healing performance with high antimicrobial properties	Potential toxicity of borax	Drug delivery	[60]
PVA/CS/GO	Freeze–thaw cycle	Dual effects: electronic drug release and tissue repair	Potential cytotoxicity at low CS concentration	Drug delivery	[61]
rGO, PDA, ZIF-PVA and CS	Bidirectional freezing and phase separation method	Enhanced mechanical properties with low hemolysis rate	Minor reduction in wound healing area observed with applied irradiation	Wound treatment	[62]
PVA and CNT	A freeze-casting combined with compression annealing and salting-out (FCAS) approach	Excellent mechanical properties with high water content	Minimal investigation into long-term stability under physiological conditions	Tissue engineering	[63]
PVA	Salting-out after syneresis	High mechanical strength combined with superior optical transparency	Increased crystallinity potentially leading to reduced hydration within surface networks	Tissue engineering	[64]

β-CD: β-cyclodextrin; PAA: poly acrylic acid; CS: chitosan; PVA: polyvinyl alcohol; CNT: carbon nanotube; rGO: Reduced graphene oxide; PDA: polydopamine.

### 2.1. PVA Hydrogels Synthesized by Repeated Freezing/Thawing Cycles in Aqueous Solutions

**Figure 1:**
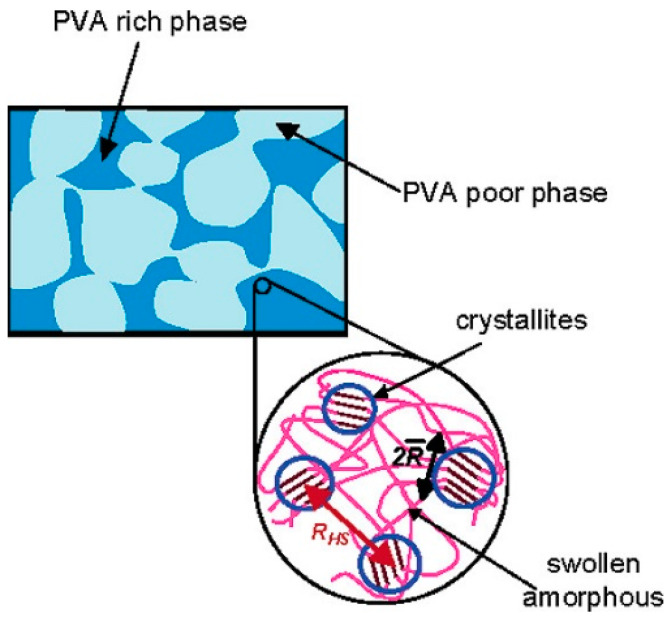
Schematic of the bicontinuous architecture of PVA hydrogels, achieved through freeze/thaw cycles, consists of two distinct phases: a PVA-rich phase and a PVA-poor phase. The circles in the inset depict, in a schematic manner, the hard-sphere model employed to fit the small-angle neutron scattering (SANS) equations to the experimental data. The figure is adopted from the ref. [69]. Copyright © 2005, American Chemical Society.

One of the most well-established methods for fabricating PVA hydrogels is the repeated freezing and thawing (FT) technique [65,66]. This approach forms physical cross-links through the crystallization of PVA chains in an aqueous solution [67]. The FT process has been the focus of extensive research and refinement due to its ability to produce hydrogels with highly customizable properties. The procedure consists of several key stages: supercooling, nucleation (thawing), crystal growth, and network aging [68]. As the solution freezes, the water separates and forms ice crystals, pushing the PVA chains closer together and promoting phase separation [69]. This results in the rapid formation of crystalline domains within the PVA, which act as physical cross-links and stabilize the hydrogel structure. For example, Rosa Ricciardi et al. [69] explore the structural properties of PVA (polyvinyl alcohol) hydrogels obtained at varying numbers of freeze/thaw cycles, with a particular focus on mesoscopic length scales. This investigation uses small-angle neutron scattering (SANS) measurements to gather detailed structural information within the range of a few tens of nanometers. This allowed researchers to gain valuable information about the hydrogel’s structural characteristics within the specified scale. The SANS data, depicted in Figure 1, did not align with reasonable fitting parameters when applied to the existing model. This discrepancy is likely due to the minimal fraction of crystallites within the gel, which exhibit a smooth surface, making them incompatible with a model that assumes polydisperse, homogeneous spherical crystallites interacting through a hard-sphere potential. Moreover, the scattering from the amorphous chains that link these crystallites (often referred to as tie-chains) can be neglected. This observation aligns with previous findings regarding the structure of GEL-1 samples. Interestingly, PVA hydrogels that undergo only a single freeze/thaw cycle exhibit reduced crystallinity compared to other samples. These gels also demonstrate suboptimal mechanical properties, including lower mechanical strength and shear elastic modulus, which is indicative of their weaker structural integrity.

Additionally, the cross-links are primarily driven by hydrogen bonding between the PVA chains, which creates a porous network capable of absorbing large quantities of water without compromising its mechanical integrity [5,51,70]. Several factors influence the crystallinity and porosity of the hydrogel, including the concentration of PVA, freezing and thawing temperatures, duration of freezing, and the number of FT cycles [71]. Other factors, such as the presence of solutes or additives, also impact the mechanical characteristics, swelling behavior, and viscoelasticity of the hydrogel [72].

In addition to these experimental settings, molecular properties of PVA, including molecular weight, degree of hydrolysis (DH), tacticity (the arrangement of monomer units), and critical concentration in solution, play a key role in determining the final properties of the hydrogel [73]. These factors must be precisely controlled to design hydrogels that meet the specific requirements of a given application. The beauty of the FT method lies in its simplicity, cost-effectiveness, and eco-friendly nature, making it especially appealing for biomedical applications.

**Figure 2:**
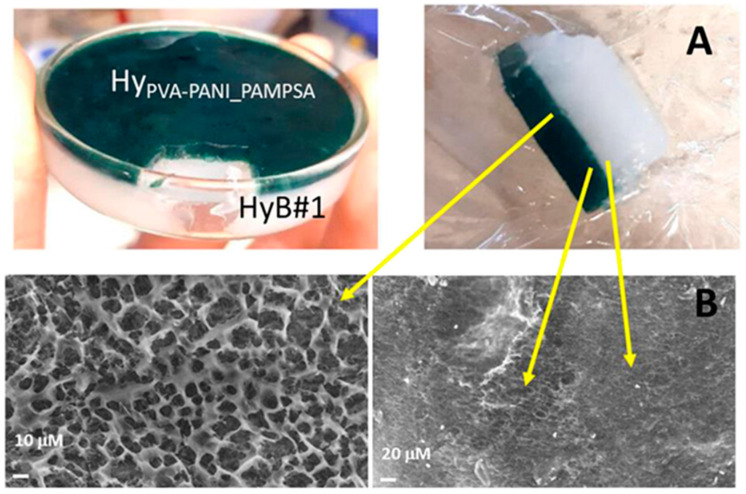
(**A**) Representation of the self-healing double-layer hydrogel HyPVA–PANI_PAMPSA/HyB#1. (**B**) Scanning electron microscopy (SEM) images of the lyophilized hydrogel. The figure is adopted from [76]. Copyright © 2024, American Chemical Society.

The FT technique can also be combined with other natural and synthetic polymers, as well as proteins, to create hydrogels with enhanced properties [41,74]. For example, incorporating materials like gelatin, collagen, or chitosan with PVA has been shown to improve cell adhesion, promote tissue growth, and enable controlled release of bioactive compounds [75]. Additionally, PVA hydrogels produced through FT cycles exhibit remarkable self-healing abilities [76], a crucial property for applications in tissue engineering [77], where hydrogels must recover from mechanical stress and retain their functionality. For example, Giada D’Altri et al. [76] developed a self-healing PVA–H_2_SO_4_ hydrogel using a freeze–thaw process. The structure consisted of two layers: the first layer (HyB#1) was pure PVA, while the second (HyPVA–PANI/PAMPSA) contained PVA combined with conductive PANI_PAMPSA polymers, which gave it a deep green color. The hydrogel, formed after six freeze–thaw cycles, had a total thickness of 9 mm, with the green layer representing PVA/PANI_PAMPSA and the white layer representing PVA shown in Figure 2. Self-healing was confirmed through optical and SEM microscopy, which showed a perfect bond between the layers. The hydrogel revealed remarkable mechanical properties, with a tensile strain of 53 ± 4% without splitting during stretching tests.

PVA hydrogels formed via FT cross-linking are highly prized in the biomedical and pharmaceutical sectors due to their high purity and the mild conditions required for their preparation [78,79]. The versatility of the FT method allow us to create a drug delivery systems with highly controlled pore sizes and surface properties, making them ideal for therapeutic applications [80]. By adapting the molecular weight of PVA and optimizing the freezing and thawing conditions, hydrogels can be engineered to provide controlled release of drugs through mechanisms such as swelling, diffusion, or erosion [81]. Further modifications to the cross-linking density can regulate the release rate, while the incorporation of proteins or other bioactive molecules can enhance the therapeutic effectiveness of the system [82]. Although FT hydrogels typically adopt semi-crystalline structures, their increased stiffness can limit their use in soft tissue applications, where modulus values typically range from 1 to 100 kPa. However, by adjusting the FT conditions, it is possible to create hydrogels with higher modulus values (>100 kPa) to mimic the mechanical properties of more robust tissues, such as tendons or cartilage [83]. For softer tissues, such as skin, where modulus values range from 1 Pa to 10 kPa, modifications to the FT process are necessary to achieve the desired flexibility [84].

To address the need for ultra-soft hydrogels, a modified approach known as the suppressed freezing/thawing (SFT) technique was developed [41]. This technique involves adding anti-freezing salts to the precursor solution, which lowers the freezing point below the cryogenic temperature. The existence of these salts inhibits the growth of ice crystals during freezing, preventing the formation of a rigid crystalline structure. Instead, an amorphous network of PVA chains forms, characterized by both free and hydrogen-bonded –OH groups. This structure results in hydrogels that closely resemble the softness and flexibility of skin and other soft tissues. These hydrogels boast exceptional properties, including a low Young’s modulus (<10 kPa), significant stretchability (~600%), high transparency (~92%), rapid self-healing (<0.3 s), and elevated ionic conductivity (2.94 S m^−1^ at 20 °C). Additionally, they maintain impressive anti-freezing properties (down to −58 °C) and superior water retention, losing only about 10 wt.% water compared to conventional cryogels, which typically lose around 74 wt.% [84].

**Figure 3:**
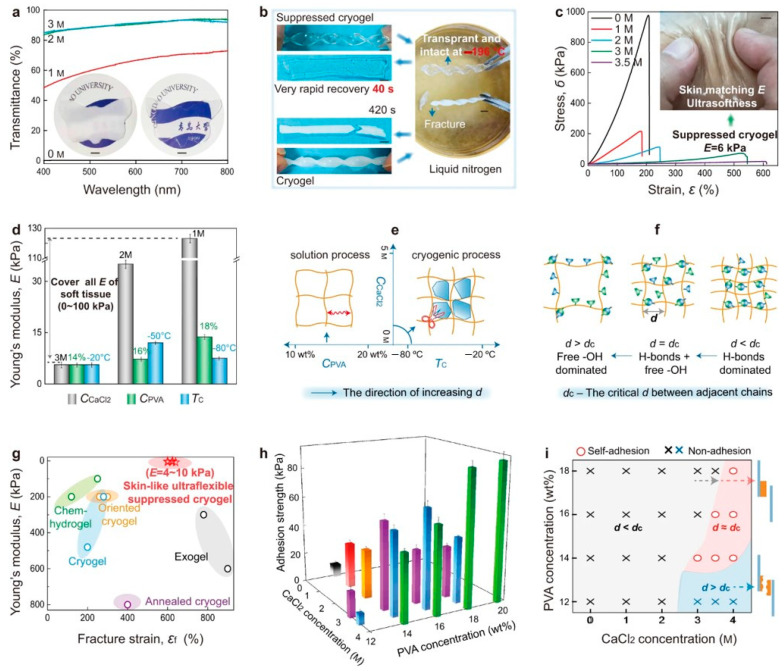
(**a**) Transmittance of 2 mm thick cryogels and suppressed cryogels (inset: images of opaque cryogels and translucent suppressed cryogels (3 M)); (**b**) Cryogels and suppressed cryogels immersed in liquid nitrogen (−196 °C) and returned to ambient temperature (24 °C); (**c**) Stress–strain curves and image of flexible suppressed cryogels (ultra-low Young’s modulus) conforming to skin; (**d**) Variation in Young’s modulus with CaCl_2_ concentration, PVA concentration, and cryogenic temperature; (**e**,**f**) Schematic of the effects of CPVA, CCaCl_2_, and Tc on cryogel microstructure during the FT process and variation of –OH group bonding with chain distance; (**g**) Comparison of Young’s modulus and fracture strain across various PVA hydrogels and cryogels; (**h**,**i**) Variation in adhesion strength with CPVA and CaCl_2_ at Tc = −20 °C. Error bars represent standard deviation (*n* = 6); scale bars: 5 mm in (**a**–**c**). The figure is adopted from the ref. [84] which is under Creative Commons Attribution 4.0 International License (http://creativecommons.org/licenses/by/4.0/, accessed on 10 August 2023).

The SFT hydrogels also demonstrate remarkable optical properties, with a transmittance of 91.6% at a wavelength of 600 nm, which contrasts with the opaque appearance of the FT hydrogels [84]. When exposed to extremely low temperatures (−196 °C, liquid nitrogen), SFT hydrogels retain both their transparency and form, while FT hydrogels become brittle and opaque. Upon thawing at 24 °C, the SFT hydrogels recover flexibility in just 40 s, while FT hydrogels require up to 420 s to regain their properties. These remarkable characteristics were vividly demonstrated by Zhang et al. [84], highlighting the potential of the SFT method to produce ultra-soft, highly functional hydrogels for a range of applications, from advanced biomedical devices to flexible electronics (Figure 3).

### 2.2. Non-Cryogenic Physical Gelation of PVA

PVA stands out as a versatile polymer renowned for its ability to spontaneously form a three-dimensional network when dissolved in aqueous solutions and organic solvents [1,12]. This unique behavior is driven by the thermodynamic principles of phase separation, where spinodal decomposition and gelation occur simultaneously. Essentially, as the PVA solution transitions from a liquid to a solid state, the polymer chains interact to create a stable hydrogel network [85,86]. The success of this transformation hinges on a fine balance between the interactions among polymer chains, solvent molecules, and environmental factors such as temperature.

What makes PVA gelation particularly appealing is its simplicity. Unlike other hydrogel systems, PVA does not require the addition of external cross-linkers, making its synthesis straightforward, cost-effective, and environmentally friendly [87,88]. To initiate gelation, a PVA solution is decanted into a mold and left at room temperature for several days. During this period, water slowly evaporates, causing the polymer chains to entangle more tightly [89]. Intra- and intermolecular hydrogen bonds form as the water content diminishes, reinforcing the network and enabling the solution to transform into a solid gel [90]. This process can be accelerated by incorporating miscible solvents like alcohols, which aid in water removal by disrupting the hydrogen bonds between water and PVA, encouraging stronger polymer-polymer interactions [91].

PVA gelation also exhibits a fascinating ability to increase its crystallinity under certain conditions [5,91,92]. For instance, when exposed to non-solvent aliphatic alcohols, the hydrogen bonds between water and PVA are replaced by stronger intermolecular hydrogen bonds between PVA chains [93,94]. This transition enhances the rigidity and stability of the polymeric network, making it particularly useful for stabilizing electrospun PVA fibers, which would otherwise dissolve in water. To further streamline the dehydration process, drying agents, such as ethylene glycol, acetonitrile, or glycerol, can be employed, ensuring a more efficient and controlled hydrogel formation.

Moreover, PVA can undergo in situ crystallization in mildly alkaline conditions, such as a 4% *w*/*w* sodium hydroxide (NaOH) solution [95]. In this environment, hydroxide ions (HO^−^) deprotonate the hydroxyl (-OH) groups of PVA, resulting in negatively charged oxygen atoms (O^−^) that interact with sodium cations (Na^+^) [96]. This interaction aligns the polymer chains into crystalline domains. Simultaneously, the hydrolysis of acetate moieties further enhances the crystallization process. When combined with a two-phase aqueous environment, this system can be employed in advanced techniques like 3D printing, where layer-by-layer adhesion occurs. The addition of sodium sulfate (Na_2_SO_4_) induces a salting-out effect, strengthening hydrogen bonds within the network and ensuring the structural fidelity of printed 3D objects [97]. Another powerful technique for PVA gelation is electrospinning, which transforms entangled PVA solutions into fibers with diameters ranging from nanometers to micrometers [98,99]. Several factors, including polymer concentration, PVA tacticity, and the tip-to-collector distance, are optimized to control the fiber characteristics [100]. Raising the applied electrical field or introducing additives like aluminum chloride (AlCl_3_) reduces the fiber diameter, creating ultra-fine structures [101]. These electrospun fibers exhibit immense potential in biomedical applications, including wound dressings, biosensors, tissue engineering scaffolds, cancer therapies, and filtration technologies [102,103].

### 2.3. Chemical Cross-Linking of PVA

Chemical cross-linking transforms PVA into a highly stable and functional material, unlocking its potential for diverse applications [104,105]. These include membrane technologies (such as pervaporation membranes and reverse osmosis systems), food packaging, desalination, wound dressings, drug delivery systems, and fuel cells [106]. The core principle of chemical cross-linking is the formation of covalent bonds between PVA molecules, enhancing the polymer’s mechanical strength, stability, and resistance to dissolution [107]. A notable result of this process is the reduction of PVA’s hydrophilicity, achieved by decreasing the number of free hydroxyl (-OH) groups available for water binding [108,109]. When cross-linking agents are introduced, they form a robust network within the gel matrix, creating a structure that no longer dissolves in water or other solvents [29,44,110]. However, these hydrogels retain their ability to absorb significant amounts of water or small molecules via swelling. The cross-linking process typically targets the hydroxyl groups on PVA chains, utilizing a variety of methods [111]. Traditional approaches include esterification, etherification, carbamation, and radical polymerization initiation. In contrast, modern techniques such as click chemistry, bioconjugation, and dynamic bond formation provide innovative pathways for designing PVA hydrogels with customized properties tailored to specific applications [112].

Selecting the right cross-linking agent is crucial for achieving the desired hydrogel characteristics. Commonly used agents include aldehydes (formaldehyde, glyoxal, glutaraldehyde), urea oligomers, dicarboxylic acids, epichlorohydrin, and borate compounds. For instance, formaldehyde and glutaraldehyde react with PVA’s hydroxyl groups to create acetal or hemiacetal bridges, which serve as cross-linking points [113,114]. By adapting the concentration of the cross-linker and the PVA solution, properties such as mechanical strength, water resistance, and permeability can be finely tuned [115]. An exciting application of chemical cross-linking is the development of gradient PVA membranes. These membranes feature a cross-linking gradient, where the degree of cross-linking decreases from the surface to the interior [116]. This can be achieved by surface cross-linking a cast PVA solution with glutaraldehyde. Controlled reaction times and cross-linker concentrations enable precise adjustments to membrane permeability and selectivity, making them ideal for applications like cell encapsulation and bio-separation.

The functionalization of PVA’s hydroxyl groups further expands its versatility [117]. Reactions such as esterification, acetalization, amination, sulfonation, and azidation can enhance the polymer’s properties for specific applications [118]. PVA hydroxyl groups also act as active sites for graft polymerization, broadening the scope of potential functionalities. A particularly intriguing method involves incorporating monocarboxylic acids like HCOOH, CH_3_COOH, CH_3_CH_2_COOH, or butyric acid during PVA dissolution in dimethyl sulfoxide (DMSO) at elevated temperatures [119]. In this system, DMSO prevents PVA chains from interacting prematurely, while monocarboxylic acids facilitate hydrogen bond formation, leading to the creation of a robust network. These hydrogels exhibit impressive mechanical strength, tensile elongation, and toughness. The addition of formaldehyde further enhances their stability by increasing hydrophobicity and crystallinity. Polycarboxylic acids offer another promising cross-linking route. Reactions between PVA hydroxyl groups and dicarboxylic acids yield stable 3D networks with multiple ester linkages [120]. These networks balance hydrophilic and hydrophobic properties, making them suitable for applications like drug delivery and separation processes. Citric acid, tartaric acid, L-malic acid, and sebacic acid are among the commonly used cross-linkers in this category. In aqueous solutions, a borate complex forms when PVA is near its overlap concentration [121]. This complex is stabilized by sodium cations, enabling reversible sol-gel transitions. The gel’s properties depend on the balance between cross-linking stimulated by borate ions and electrostatic repulsions within the polymer. Dynamic light scattering studies reveal distinct relaxation modes that reflect the structural dynamics of the gel [122,123].

Lastly, innovative cross-linkers like quercetin, a natural flavonoid, are gaining attention. When combined with PVA in a 50:1 (*w*/*w*) ratio, quercetin enhances the hydrogel’s mechanical strength, water resistance, and antioxidant properties, making it a favorable candidate for biomedical purposes. Despite the immense potential of chemical cross-linking, challenges such as toxicity and undesirable side reactions remain. Nonetheless, chemically cross-linked PVA hydrogels continue to offer innovative solutions across medical and industrial fields, from cartilage repair to radiation shielding. The future of PVA-based materials promises even greater advancements, driven by ongoing research and development.

### 2.4. Hydrogels Synthesized Through Combined Approaches

**Figure 4:**
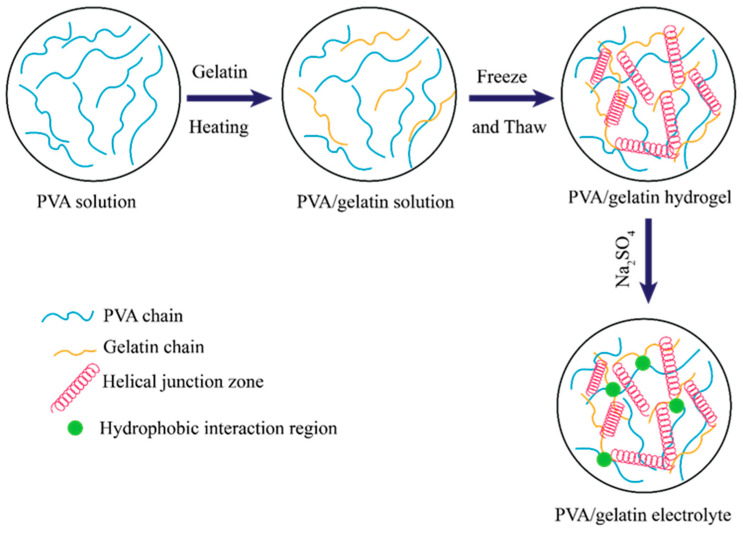
A schematic depiction of the double-network (DN) structure formation in the PVA/gelatin gel electrolyte. The first network is formed by the PVA through freeze–thaw cycling, which induces physical cross-linking. The second network is established by gelatin through the Hofmeister effect, facilitating additional interactions to enhance the gel’s structural integrity. The idea of this figure is adopted from the ref. [129].

PVA and gelatin-based three-dimensional (3D) double networks (DNs) are rapidly gaining attention as highly promising materials due to their exceptional mechanical strength and electrochemical performance [75,87]. These versatile hydrogels are synthesized through a combination of advanced techniques. The first PVA network is formed through freezing–thawing (FT) cycles, where the polymer solution undergoes alternating freezing and thawing [124,125]. This process induces physical cross-linking via hydrogen bonding, resulting in a stable and flexible PVA structure with well-defined mechanical properties and swelling characteristics [126,127]. The second network, composed of gelatin, is created using the Hofmeister effect, a phenomenon in which ions influence the solubility and structure of proteins or polysaccharides, creating a complementary, robust network [128]. Combining these two networks results in a hydrogel that excels in mechanical properties, capable of withstanding significant deformation. For example, Binbin Hou et al. [129], reported that when the gelatin/PVA ratio is set at 1/6, the hydrogel achieves a tensile strength of 1.0 MPa, an extension at break of 582%, and a fracture energy of 3.2 MJ/m^3^. It also demonstrates impressive compressive strength of 283 MPa at 95% strain. These excellent properties arise from the synergistic interactions between the PVA and gelatin networks, which together provide both structural integrity and remarkable flexibility. Increasing the gelatin content further enhances the hydrogel’s water absorption capacity, while the addition of Na_2_SO_4_ significantly boosts its conductivity. The electrochemical potential of these hydrogels was demonstrated in a supercapacitor setup, where reduced graphene oxide (rGO) was used as the electrode material and DNs served as the electrolyte gel. The supercapacitor exhibited a specific capacitance of 83.6 F/g at a current density of 1 A/g, demonstrating the hydrogel’s remarkable electrochemical properties. A similar approach, combining FT cycles and the salting-out effect, was employed to prepare PVA/gelatin hydrogels for flexible electronic applications, incorporating borax and Na_2_SO_4_ solutions, along with ethylene glycol/water mixtures. The resulting DNs exhibited exceptional mechanical performance, including a fracture tensile strain of 1281%, tensile strength of 1.12 MPa, and toughness of 7.19 MJ/m^3^ (Figure 4). Furthermore, these hydrogels demonstrated remarkable recovery properties, with the Young’s modulus and toughness recovering to 97.36% and 97.47%, respectively, after 180 min of recovery. These high recovery rates indicate not only excellent resilience but also make these hydrogels ideal candidates for applications that require repeated deformation, such as in flexible electronics and soft robotics.

In another study reported by Yuchen Zhang et al. [130] robust, tough, and elastic PVA/polyacrylamide (PAm) DNs were synthesized to replicate the properties of native articular cartilage. These hydrogels were produced through in situ polymerization, followed by treatment with various anions such as Cit^3−^, SO₄^2−^, and Cl^−^. Among these, Cit^3−^-induced networks exhibited superior performance, achieving a tensile strength of 18.9 MPa, compressive strength of 102.3 MPa, tensile modulus of 10.6 MPa, compressive modulus of 8.9 MPa, and roughness of 66.2 MJ/m^3^. These hydrogels also displayed remarkable bio-adhesion, effectively promoting chondrocyte proliferation—a crucial property in tissue engineering, where encouraging cell adhesion and growth is essential for successful tissue regeneration.

Despite its versatility, PVA’s inherent water solubility limits its practical applications [11]. To overcome this challenge [131]. Physical cross-linking forms reversible bonds between polymer chains, while chemical cross-linking creates permanent covalent bonds, ensuring greater stability. cross-linked PVA nanofiber mats, which have a porous structure, are particularly useful for applications such as filtration membranes or adsorbent materials [132]. However, PVA fibers do not cross-link effectively when exposed to formic acid or glutaraldehyde vapors and require cross-linking in solution [133]. Even then, their stability in water remains limited. Thermal treatment offers a promising solution, enabling PVA fibers to retain their properties in aqueous environments. Moreover, PVA fibers cross-linked with glutaraldehyde (GA) in organic solvents have been shown to maintain their morphology and mechanical features even after exposure to severe conditions, such as hot water or harsh acidic or alkaline environments over extended periods [5,31,129].

The hydrolytic stability of electrospun PVA/chitosan membranes can be significantly enhanced by cross-linking with GA in the presence of hydrochloric acid [134]. These membranes exhibited excellent filtration efficiency and antibacterial properties, making them ideal for use in protective masks. Additionally, PVA nanofibers cross-linked with maleic acid showed significant improvements in filtration efficiency [135]. PVA fibers partially cross-linked with GA and processed in a reactor (a long hot tube) before electrospinning demonstrated enhanced mechanical properties and thermal stability compared to those cross-linked with GA vapors alone [136]. These enhancements in thermal stability and water resistance are critical for broadening the range of applications of PVA hydrogels in challenging environments. A synergistic influence was perceived when two cross-linking agents—glutaraldehyde (GA) and ammonium persulfate—were used together to improve both water resistance and thermal stability in PVA-based hydrogels [137]. Additionally, dual-reinforcing strategies, involving chemical cross-linking with glutaric acid and incorporating reinforcing agents such as tungsten disulfide nanotubes and carboxylated multiwall carbon nanotubes, have been shown to significantly enhance the thermal and mechanical characteristics of PVA hydrogels. Other approaches, such as cross-linking with tartaric acid followed by microwave treatment or normal heating, have also led to improvements in the properties of these hydrogels [138]. However, it is important to note that certain cross-linking agents, such as GA and epichlorohydrin, can create toxic microenvironments that reduce hemocompatibility and promote thrombus formation, depending on the surface characteristics of the hydrogel.

To characterize the properties of PVA hydrogels, several advanced techniques are employed to evaluate their physical, chemical, and mechanical attributes, which are crucial for optimizing their enactment for specific applications [13]. PVA hydrogels are widely used in drug delivery systems, where they typically exhibit an initial burst release followed by a lag phase. To mitigate these effects, surface cross-linking can be introduced to modify the release kinetics. By carefully adjusting the cross-linker concentration and exposure time, it is possible to achieve the desired surface layer thickness and cross-linking density, effectively eliminating the burst release and ensuring a controlled, delayed release profile. This modification is essential for creating reproducible and predictable drug delivery systems.

The complex structures formed during cross-linking result in a variety of viscoelastic responses when subjected to large-amplitude oscillatory shear (LAOS) [139]. PVA/borax-based physical gels, with temporary junction points, exhibit shear thinning at large strains, followed by strain stiffening as polymer segments stretch between cross-links. In contrast, chemically cross-linked PVA/hyaluronic acid (HA) networks exhibit intra-cycle strain stiffening and shear thickening at larger strains, with minimal frequency distinction in the viscoelastic moduli in the linear range. These unique rheological behaviors are crucial for understanding the mechanical properties of hydrogels, particularly in uses concerning flexible and dynamic systems, including soft robotics or wearable electronics.

## 3. Biomedical Applications of PVA-Based Composite Hydrogels

**Scheme 1:**
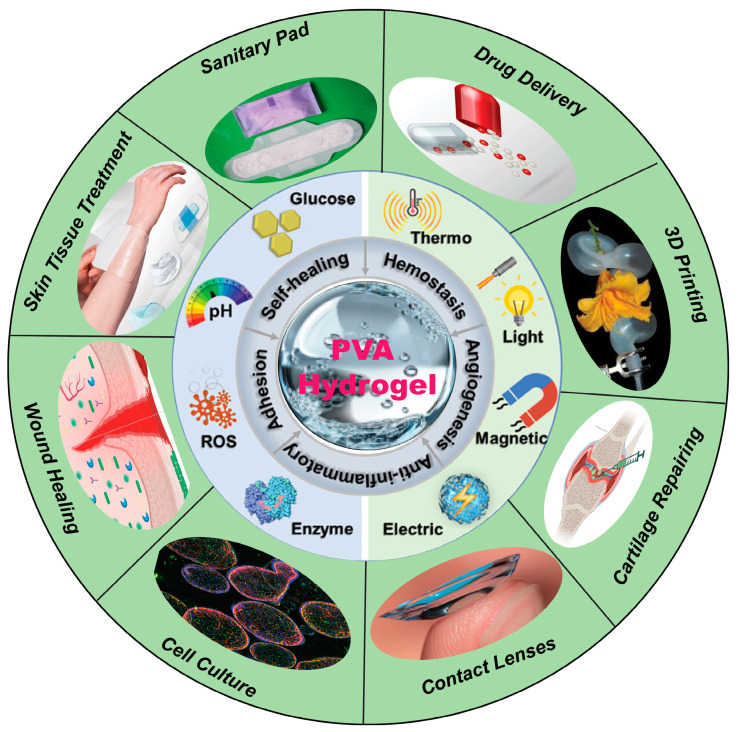
Various biomedical applications of PVA-based hydrogels.

Owing to their remarkable mechanical strength, favorable biocompatibility, and easy biodegradability, PVA, PVP, and their composite biomaterials have been extensively studied and utilized across a broad spectrum of cutting-edge purposes in biomedical, pharmaceutical, food, and commercial sectors [140]. Among these, the areas of greatest interest include tissue engineering (such as articular cartilage, bones, artificial pancreas, artificial skin, and vascular devices), drug delivery, wound and burn treatments, and ophthalmic applications (including artificial corneas, contact lenses, and synthetic vitreous bodies) [51,52,141]. Various biomedical applications of PVA-based composite hydrogels are demonstrated in Scheme 1.

In addition to the established applications of PVA-based hydrogels, emerging trends indicate their potential in less-explored biomedical fields. For example, PVA-based hydrogels show promise as bio-inks for 3D bioprinting, enabling the fabrication of complex tissue constructs with high precision for regenerative medicine. Their adaptability and mechanical tunability make them ideal for injectable hydrogel systems, which are gaining traction for minimally invasive therapies in tissue repair and localized drug delivery [142]. Furthermore, these hydrogels can be engineered into smart biosensors for real-time health monitoring and diagnostic applications, offering sensitivity to physiological changes such as glucose levels or pH. Another area of interest is their use in organ regeneration, where PVA-based hydrogels could serve as scaffolds mimicking the extracellular matrix, promoting cellular growth and tissue integration. By exploring these emerging areas, the vast potential of state-of-the-art PVA-based hydrogels in revolutionizing biomedical technologies and advancing personalized healthcare solutions. However, recent advancements in PVA-based hydrogels have led to the development of ultra-soft magnetic miniature robots capable of double-modal locomotion with switchable forms. By adjusting the magnetic field, these flexible robots can transition between stretching and contracting shapes, enabling diverse movement patterns [143]. In their bar-stretch state, they perform forward swinging motions, while in a spherical state, they execute tumbling movements [143]. This versatility positions them as promising candidates for real-time biomedical applications, such as targeted drug delivery and minimally invasive procedures, where adaptability and precise control are crucial. Wenqun Li and colleagues [144] developed puerarin-loaded PEG-PE micelles to enhance the pharmaceutical properties of puerarin. These micelles demonstrated an improved anti-apoptotic effect and a better pharmacokinetic profile, suggesting their potential for more effective therapeutic applications.

### 3.1. Tissue Engineering and Regenerative Medicine

Tissue engineering and regenerative medicine are rising and dynamic fields, merging various disciplines to create artificial substitutes aimed at restoring, maintaining, and enhancing tissue functionality in cases of severe injuries that are otherwise difficult to treat [145]. Biomimetics has been fundamental to this area, as it emphasizes observing natural tissues to derive principles that can be applied in developing artificial tissues closely resembling their biological counterparts. The primary components in tissue engineering include scaffolds that imitate the extracellular matrix, cells, and growth factors [27,146]. Scaffolds are three-dimensional structures with large surface areas and interconnected pores, serving as templates for seeding regenerative cells. These scaffolds, due to their chemical and physical properties, support cell attachment, proliferation, and new tissue formation, while the scaffold itself degrades over time [147,148]. Hence, materials chosen for scaffold fabrication must be biocompatible and biodegradable, with mechanical properties similar to natural tissues.

Significant research has been conducted over recent decades on producing scaffolds using biocompatible and biodegradable polymers for tissue engineering [149]. Among these, PVA and PVP stand out due to their flexibility in design, allowing customization to meet specific requirements, such as high-water content, tissue-like elasticity, and biocompatibility [150]. Studies on PVA hydrogels have shown that they are promising candidates for tissue engineering. Even with basic cryogenic gelation methods, PVA hydrogels form porous structures capable of absorbing surrounding fluids, demonstrating the potential to closely mimic human tissues. The use of PVA in the medical field, particularly for blood vessel disease treatment, has been well established, with applications dating back to its use in embolic agents since 1975 [151].

On the other hand, in tissue engineering, PVA-based hydrogels serve as scaffolds that mimic the extracellular matrix, providing a supportive framework for cell growth and differentiation. The biocompatibility of PVA ensures that cells adhere, proliferate, and differentiate effectively, while surface functionalization with bioactive molecules like RGD peptides enhances these interactions. Mechanical strength is critical in this application, as scaffolds must withstand physiological stresses while maintaining their structural integrity. PVA hydrogels can be cross-linked to achieve viscoelastic properties that closely resemble native tissues, making them ideal for applications such as cartilage or skin regeneration. Furthermore, the biodegradability of PVA-based scaffolds can be tuned by incorporating degradable linkages or blending with polymers like polycaprolactone, ensuring that the scaffold degrades in tandem with new tissue formation, eliminating the need for surgical removal.

Research has also explored the integration of PVA with other materials, such as chitosan, to improve functionality for treating arterial diseases [152]. For example, PVA hydrogels incorporated with chitosan have shown promising results for promoting cell adhesion and proliferation of vascular cells, which could be useful in the development of artificial blood vessels [152,153]. Enhancing the mechanical properties of PVA, PVP, and PVA/PVP biomaterials has been a key focus in research [9,154]. The inclusion of carbon nanotubes (CNTs) has been explored as a means to enhance the mechanical, electrical, and thermal characteristics of these materials [155]. CNTs are known for their excellent potential for surface modification, making them ideal for reinforcing bio-composites used in tissue engineering scaffolds, soft implants, and drug delivery systems [156]. Notably, PVA hydrogels reinforced with CNTs show improved tensile strength and tear resistance, even at low concentrations of CNTs.

**Figure 5:**
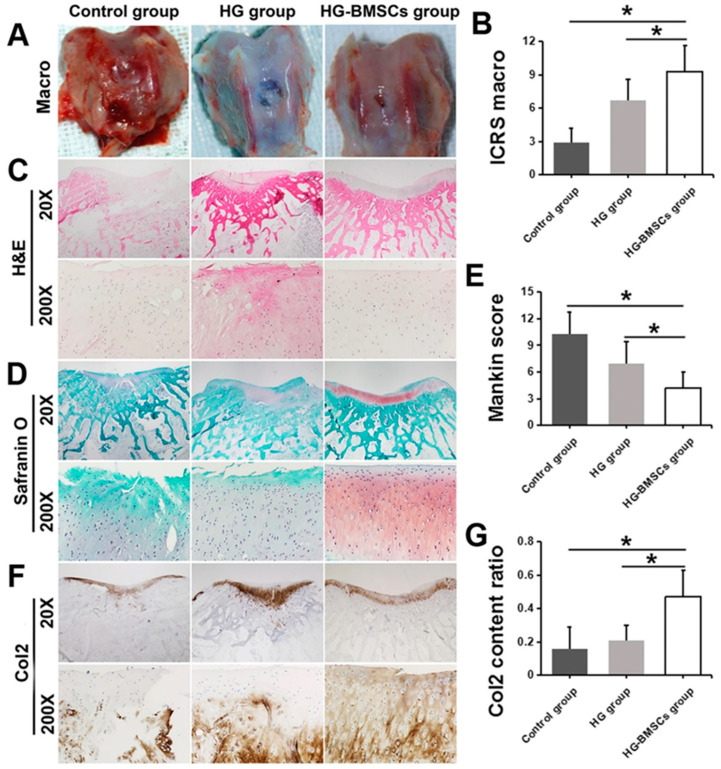
Schematic assessment of osteochondral defect repair in the control, HG, and HG-BMSCs groups at 12 weeks post-surgery through both macroscopic and histological evaluation. (**A**) Observational macro images. (**B**) ICRS scoring for macroscopic evaluation. (**C**) H&E histological staining. (**D**) Safranin O staining results. (**E**) Mankin score analysis. (**F**) Collagen type II (Col_2_) staining. (**G**) Quantification of the Col_2_ content ratio. (* represent the experimental conditions). The figure is adopted with permission from the ref. [157] which is under Creative Commons Attribution 4.0 International License (http://creativecommons.org/publicdomain/zero/1.0/, accessed on 29 May 2019).

Liangquan Peng et al. [157], investigated the combination of PVA and CS in varying ratios to enhance the mechanical properties and biocompatibility of the hydrogel while addressing the limitations associated with using a single material. This innovative PVA/CS porous hydrogel was evaluated for its physical and chemical characteristics, mechanical performance, and its ability to support the culture of rabbit bone marrow mesenchymal stem cells (BMSCs) in vitro [157]. Both the hydrogel alone and in combination with BMSCs were utilized for cartilage repair and regeneration in a rabbit osteochondral defect model to assess their efficacy in an in vivo setting. Three weeks post-surgery, the control group exhibited a clear osteochondral defect with no signs of healing. In contrast, the hydrogel group showed partial repair of the defect with a white, organized, yet incomplete surface. The hydrogel-BMSC group demonstrated superior healing, with the patch seamlessly integrating into the surrounding healthy cartilage. The repaired articular cartilage (AC) displayed a smooth, well-organized surface with uniform color and contour resembling normal AC. In the ICRS macroscopic evaluation, the integration with the border zone was significantly greater in the HG-BMSC group compared to the hydrogel and control groups (9.3  ±  2.3 vs. 6.7  ±  1.9 vs. 2.9  ±  1.3, respectively; *p*  <  0.05) [157] (Figure 5).

The design of scaffolds that foster the development of specialized cells into the desired tissue or organ is central to the advancement of tissue engineering [158]. Scaffolds serve as an initial framework for tissue regeneration, with hydrogels playing a crucial role due to their similarity to the extracellular matrix [158,159,160]. In bone regeneration, polymeric hydrogels are generally utilized for both injection and implantation approaches [161,162]. Research has also shown promising results using chondrocyte-derived progenitor cells for cartilage regeneration, particularly in knee injuries, representing superior chondrogenic potential compared to mesenchymal stem cells [163,164,165]. PVA hydrogels have shown significant promise in knee meniscus repair, with studies assessing cell viability within PVA scaffolds indicating excellent compatibility [166]. Additionally, the incorporation of PVA with other bioactive agents, such as hypericum perforatum oil (HPO), has been explored for enhancing skin tissue regeneration. HPO has known antioxidant and anti-inflammatory properties, and when added to PVA/chitosan hydrogels, it improves material characteristics like hydrophilicity and flexibility, promoting cell growth and viability.

Furthermore, research into quercetin-loaded PVA/chitosan hydrogels has highlighted their antibacterial potential and low toxicity, making them suitable for tissue engineering applications [167]. The scaffolds’ ability to inhibit hemolysis and support cell growth further underscores their applicability in regenerative medicine [168]. The repair and regeneration of cartilage defects remain a major challenge in tissue engineering. Hydrogels, such as scaffolds, are ideal for such applications due to their adjustable properties. Both natural and synthetic polymers play critical roles in promoting cell attachment, migration, and differentiation, which are essential for cartilage regeneration. Studies involving composites of PVA reinforced with hydroxyapatite and Se-doped TiO_2_ nanoparticles have demonstrated improved mechanical properties and cellular compatibility, with potential applications in bone and cartilage tissue engineering [169].

In conclusion, while traditional hydrogels are widely used for scaffolding, there is an ongoing need to develop materials that better mimic the dynamic and heterogeneous nature of native tissues, addressing current limitations in tissue engineering applications.

### 3.2. Wound Dressing

Wounds, resulting from physical injuries or traumas, can damage both internal and external tissues, particularly the skin, thereby impairing its structural integrity and normal function [20,170]. The wound healing process typically progresses through four distinct stages: hemostasis, inflammation, proliferation, and remodeling [20,171,172]. To facilitate effective healing, an ideal wound dressing must maintain a moist environment conducive to epithelial cell growth, safeguard against external contaminants, manage wound exudates, provide flexibility, be biocompatible, allow adequate oxygen exchange, and minimize skin adherence [173,174]. PVA hydrogels exhibit numerous properties that make them well-suited for wound dressing applications, including promoting autolytic debridement and assisting in the removal of necrotic tissue [175,176,177]. Their exceptional water retention capacity, coupled with their insolubility in water, expedites the healing process by absorbing wound fluids and reducing the likelihood of infections. Additionally, PVA hydrogels enhance wound closure by facilitating the migration of fibroblasts. However, the hydrophilic nature of PVA ensures excellent water retention, which aids in creating an optimal wound-healing environment. Their inherent biocompatibility minimizes immune responses, while functionalization with antibacterial agents like silver nanoparticles or growth factors further enhances their efficacy in infection control and tissue regeneration. Additionally, PVA hydrogels can be engineered to exhibit appropriate mechanical properties, ensuring durability and flexibility in dynamic environments, such as wounds located on joints or areas subject to movement. To address biodegradability, blending PVA with polymers such as chitosan or polylactic acid introduces controlled degradation, making the hydrogels suitable for temporary applications without requiring removal.

The biocompatibility, biodegradability, and extracellular matrix-like structure of PVA hydrogels position them as excellent candidates for next-generation wound care [178]. Unlike conventional bandages that often adhere to the wound site, hindering recovery, PVA hydrogels provide a controlled delivery mechanism for bioactive substances. This system ensures gradual and localized release, allowing for efficient and patient-friendly wound management. Moreover, these hydrogels are characterized by their ease of degradation and non-toxic nature, further endorsing their application in wound care [179]. Incorporating bioactive compounds, such as diphlorethohydroxycarmalol derived from the brown alga Ishige Okamurae, into PVA hydrogels enhances cell proliferation and tissue regeneration, accelerating the healing process [180]. Studies reveal that PVA/DPHC hydrogels exhibit significant antimicrobial activity, achieving approximately 99% reduction in Staphylococcus aureus and Pseudomonas aeruginosa in ASTM E2149 assessments [181,182]. Importantly, these hydrogels have demonstrated non-cytotoxicity toward NHDF-Neo and HaCaT cells, as verified by MTT assays and fluorescein diacetate (FDA) fluorescence analysis. In vivo experiments conducted on ICR mice have shown remarkable wound closure rates, with 75% healing within seven days and full recovery by day 14 [183].

The utilization of bioactive compounds within hydrogels represents an innovative strategy that surpasses traditional dressings, which are often embedded with antibiotics [184]. While antibiotic-infused dressings risk fostering bacterial resistance and diminishing treatment efficacy, bioactive compound-enriched PVA hydrogels provide a safer alternative. These compounds, offering antibacterial, antioxidant, and anti-inflammatory properties, improve wound management without the drawbacks associated with antibiotics [185,186]. For example, honey, rich in phenolic acids, flavonoids, carotenoids, and organic acids, delivers notable antibacterial and antioxidant benefits for wound healing [187]. However, direct application of honey may cause discomfort and limit its effectiveness. The study by Shamloo et al. [188] highlights that increasing honey concentrations in PVA hydrogel composites boosts antibacterial efficacy, enhances cell viability, and accelerates healing. Nonetheless, the addition of honey can compromise the mechanical durability of the hydrogel and accelerate its degradation, although the composite retains sufficient strength for clinical use in wound care [188].

**Figure 6:**
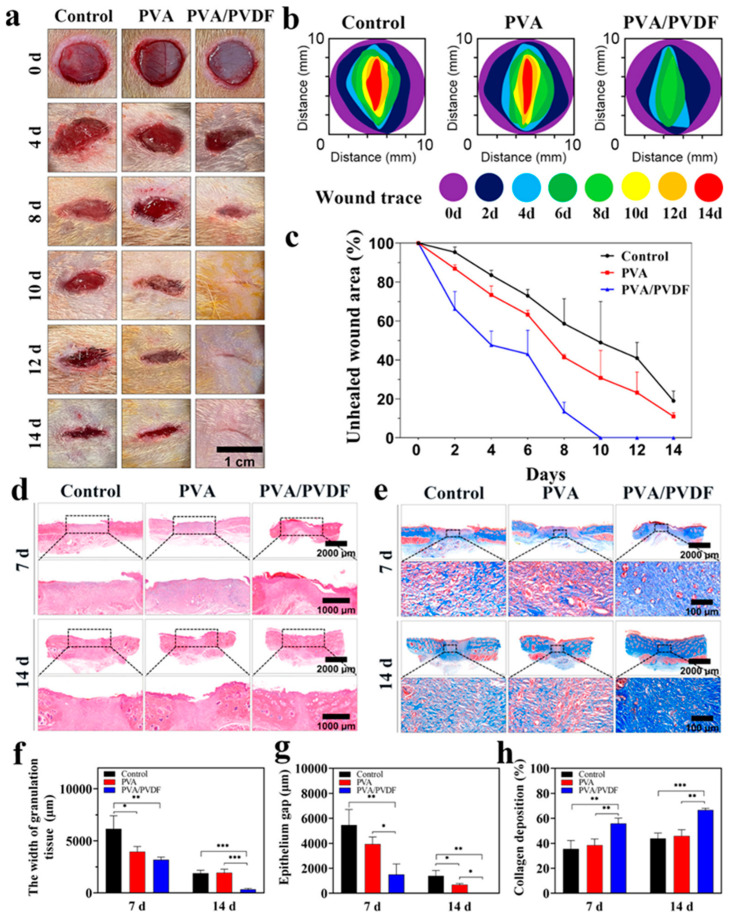
Wound healing response in diabetic SD rats. (**a**) Typical images of wounds in diabetic SD rats at various time intervals, comparing the control group, PVA hydrogel group, and PVA/PVDF composite hydrogel group. (**b**) Analysis of wound progression in different groups, observed every two days post-surgery. (**c**) Graphs showing the ratio of remaining wound area to initial wound area over time for each treatment group. (**d**) Representative H&E-stained skin tissue images from days 7 and 14. (**e**) Representative Masson’s trichrome-stained skin tissue images from days 7 and 14. (**f**,**g**) Statistical analysis of granulation tissue width and epithelial gap using H&E staining. (**h**) Statistical evaluation of collagen accumulation across various groups. (*, **,*** represent the experimental conditions). This figure is adopted from the ref. [189]. Copyright © 2022, American Chemical Society.

Limin Wang et al. [189] developed a self-sustained piezoelectric composite hydrogel made of PVA/PVDF by using a specialized preparation technique that combined freezing/thawing, solvent replacement, annealing, and swelling. The interaction between molecules in the PVA/PVDF system was strengthened during the hydrogel formation, aiding in the creation of the electroactive *β*-phase of PVDF. The hydrogel’s mechanical properties, moisture absorption, and permeability were enhanced by fine-tuning its crystalline structure and three-dimensional cross-linking network. The biocompatibility and cell behavior modulation of the composite hydrogel were evaluated, and its application in wound healing was further tested in vivo using a full-thickness skin defect model in diabetic rats [190]. The mechanical energy produced by the rats’ movements was converted into electrical energy, and the piezoelectric stimulation from the hydrogel on the wound area was used to restore the electrical microenvironment, facilitating wound healing. In the diabetic rat model, this local piezoelectric stimulation, similar to the effects observed in vitro, was continuously applied to the wound bed. As shown in Figure 6a [189], the wound area was monitored and measured at different time intervals.

Visually, none of the rats exhibited severe redness, irritation, or signs of infection. While untreated wounds remained dry and scabbed, the wounds treated with PVA and PVA/PVDF composite hydrogels were kept in a moist state. Starting from day 2, the wound healing rate was considerably faster in the PVA/PVDF composite hydrogel group compared to the others as illustrated in Figure 6a–c [189]. By day 8, the residual wound area in the PVA/PVDF composite hydrogel group was 13.5%, which was notably lower than in the PVA hydrogel group (41.4%) and the control group (58.6%). By day 10, the PVA/PVDF composite hydrogel-treated wounds were fully healed, whereas the PVA hydrogel and control groups still showed unhealed areas (30.8% and 48.9%, respectively). On day 14, wounds in the PVA hydrogel and control groups remained partially unhealed, with 11.0% and 19.0% residual wound areas, respectively. Additionally, wound closure analysis (Figure 6) showed that the PVA/PVDF composite hydrogel group had a significantly higher closure rate compared to the other groups, indicating that the PVA/PVDF piezoelectric hydrogel could greatly accelerate the healing process in diabetic rats. This approach, based on piezoelectric activity, holds promise for new treatments in chronic wound healing [190]. however, integrating natural bioactive compounds with PVA hydrogels offers significant promise for enhancing wound care technology. Plant-based extracts like Lawsonia inermis have demonstrated exceptional potential in this domain, exhibiting strong antibacterial and antioxidant properties. The findings of Khan et al. underscore the efficacy of this approach, with over 80% of wounds treated utilizing a PVA/chitosan hydrogel comprising Lawsonia inermis extract achieving full recovery within ten days. Such outcomes highlight the transformative potential of PVA hydrogels as delivery systems for bioactive agents, paving the way for advanced therapeutic solutions in wound management as seen in Figure 6d,e [189].

### 3.3. Drug Delivery

Drug-loaded hydrogel materials are predominantly employed in temporary bio-scaffolds compared to permanent replacements [190,191,192,193]. However, some investigations have demonstrated the use of PVA-based hydrogels containing anti-inflammatory agents, such as ibuprofen, diclofenac, or ketorolac, to repair cartilage lesions [194]. These hydrogels also effectively minimize postoperative infections in implant surgeries. Controlled drug release mechanisms have been realized by incorporating solid inclusion complexes like ibuprofen within β-cyclodextrin or through the addition of compounds such as vitamin E and cetalkonium chloride [195]. Beyond anti-inflammatory agents, certain PVA hydrogels have integrated components that stimulate cartilage regeneration by enhancing chondrocyte growth in the porous structure of the hydrogel [196]. For instance, calcium ions (Ca^2⁺^) have been shown to foster cellular proliferation and have been successfully utilized in cartilage repair using PVA hydrogels [197]. Similarly, hyaluronic acid (HA) has proven effective in guiding tissue regeneration [198]. On the other hand, in drug delivery, PVA hydrogels offer a versatile platform capable of encapsulating and releasing therapeutic agents in a controlled manner. Their biocompatibility ensures the safe administration of a wide range of drugs, including proteins, peptides, and small molecules, without adverse side effects. The mechanical strength of PVA hydrogels preserves their structural integrity during drug delivery, ensuring consistent and predictable release. Cross-linking density and matrix design can be tailored to achieve specific drug diffusion profiles. Moreover, incorporating biodegradable polymers allows the hydrogel to degrade gradually, synchronizing with the drug release schedule to provide sustained therapeutic effects. For instance, PVA hydrogels loaded with anticancer drugs enable localized delivery, reducing systemic side effects and improving treatment efficacy.

PVA and its derivatives, often in the form of hydrogel microparticles and nanoparticles, have gained attention for various drug delivery systems [199,200]. For instance, studies have highlighted PVA nanoparticles encapsulated by poly(lactide-co-glycolic acid) (PLGA) microparticles and paclitaxel-loaded PVA-g-PLGA nanoparticles for treating restenosis [201]. Additionally, DNA nanocarriers prepared via modified solvent displacement methods have utilized PVA as a matrix material [202].

Since the late 1990s, PVA hydrogel nanoparticles have been employed for protein and peptide drug delivery, as evidenced by early research using water-in-oil emulsions and freeze–thaw cycles to create nanoparticles with an average diameter of approximately 675 nm [203]. These particles demonstrated an impressive protein loading efficiency exceeding 96% and exhibited a diffusion-driven release profile [203]. Subsequent advancements have refined production techniques for PVA-based nanoparticles, including methods such as salting-out, emulsification diffusion, and nanoprecipitation, tailored for specific drug delivery requirements. Further studies explored PVA’s role as a drug coating material, revealing that its degree of hydrolysis significantly influences drug release rates. For instance, partially hydrolyzed PVA promotes rapid release, while fully hydrolyzed variants provide a more controlled and sustained release [204]. Similarly, cross-linked PVA systems, developed for encapsulating model drugs like theophylline, demonstrated a correlation between polymer concentration and encapsulation efficiency [205]. Due to PVA’s low gastrointestinal absorption, it is often utilized as a non-absorbing matrix for oral drug delivery. Research has demonstrated the successful use of PVA hydrogels for veterinary applications, such as the oral delivery of antibiotics, where drug retention in target tissues like kidneys and muscles was notably high [206,207,208]. Conversely, other antibiotics did not exhibit similar delivery efficacy.

In drug delivery patches for oral cancer treatment, PVA has been incorporated to enhance mucoadhesive properties and optimize release profiles, while in nasal delivery systems, PVA combined with other polymers has provided stable formulations with effective drug dispersion [209,210,211]. Additionally, PVA coatings have been applied to microparticles for aerosolized drug delivery, significantly improving suspension stability and inhalation properties [212,213,214]. Oral drug delivery systems face challenges such as inconsistent drug concentrations in the bloodstream due to rapid metabolism. To address this, polymer-based controlled delivery systems have been developed. Hydrogels, with their porous structure and high-water content, facilitate the transport of hydrophilic drugs and enable gradual and sustained release. Drug incorporation into hydrogels can be achieved either during the polymerization process or through subsequent loading via soaking methods [215,216].

Innovative applications of PVA hydrogels include devices for continuous drug release, such as those inspired by pufferfish, which respond to stomach temperature [217]. Hydrogels have also been explored for colon-targeted drug delivery, electrically controlled release, and in situ cross-linked scaffolds for localized and sustained release. The release characteristics of PVA hydrogel nanoparticles depend significantly on factors such as the number of freeze–thaw cycles and environmental temperature [218]. For example, bovine serum albumin loaded into PVA nanoparticles demonstrated a protein loading efficiency above 96% and a temperature-sensitive release profile lasting up to 30 h [219]. Furthermore, PVA hydrogels combined with alginate have exhibited effective antimicrobial activity and biocompatibility, confirmed through fibroblast interaction studies.

**Figure 7:**
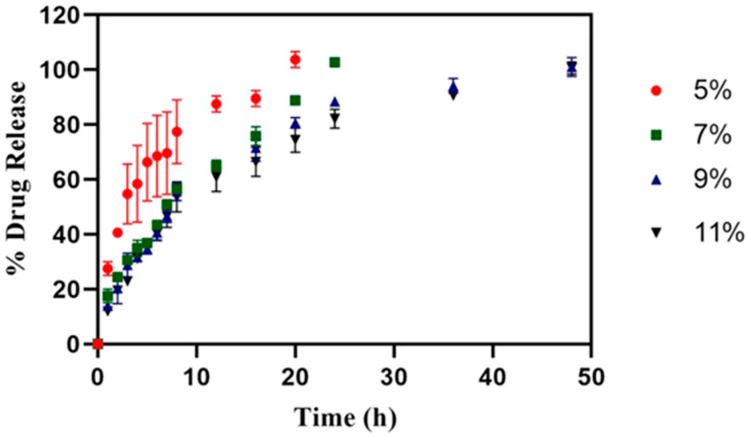
In vitro drug release of 5-NPPP loaded hydrogel. This shows the cumulative amount of 5-NPPP being released over a period of 50 h (n  =  3, *p*  <  0.05). this figure is adopted from the ref. [220] which is under Creative Commons Attribution 4.0 International License (http://creativecommons.org/licenses/by/4.0/, accessed on 14 July 2023).

Gopalakrishnan Thamilselvan et al. explored the creation of topical hydrogels that incorporated two distinct molecules, CIP (as a free drug) and 5-NPPP (within polymeric nanoparticles), as part of a combinatorial approach for treating surgical site infections (SSIs) [220]. The foundation of their work was rooted in the specific properties of the drugs, the choice of polymers, and their respective concentrations. Hydrogels were carefully designed with the optimal polymer composition that allowed for sustained drug release when applied topically, maintaining the release of both drugs over a period of 5 days, based on in vitro evaluations. In vitro release data for hydrogels containing only 5-NPPP showed sustained drug release for up to 2 days in phosphate buffer (pH 7.4). Hydrogels with low concentrations of PVA (5% and 7% *w*/*v*) achieved their peak collective drug release at 20 h and 24 h, respectively. As the polymer concentration raised (to 9% and 11% *w*/*v*), the drug release was extended, reaching 100% at 48 h, attributed to a reduction in the swelling index, as illustrated in Figure 2. Nevertheless, this release profile did not align with the desired pattern for the CIP-loaded hydrogels, which hindered the anticipated synergistic influences. To enhance the stability of 5-NPPP and achieve the necessary continued release for 5 days, the researchers aimed to develop polymeric nanoparticles for 5-NPPP and incorporate them into the hydrogels [220] (Figure 7). Hydrogels serve as versatile platforms for therapeutic delivery, offering tunable physical properties, adjustable degradability, and protection for sensitive bioactives. These materials enable controlled release mechanisms and facilitate interactions that enhance the efficacy of drug delivery systems.

### 3.4. Contact Lenses

Contact lenses serve multiple functions, such as improving vision, treating medical conditions, and enhancing cosmetic appearance [21]. They are directly placed on the cornea to correct refractive errors and promote eye health. The two main types of contact lenses are rigid and soft, and regulatory bodies like the European Union (EU) and the U.S. Food and Drug Administration (FDA) categorize them as medical devices, ensuring safety and efficacy for users [221]. Soft and rigid lenses designed for daily wear are considered moderate-risk devices (Class II) by the FDA, while extended-wear lenses are classified as higher risk (Class III). In the EU, short-term lenses are classified as Class IIa with intermediate risk, and long-term lenses as Class IIb with higher risk.

When applying contact lenses for drug delivery to the eye, special care must be taken to preserve visual clarity and physiological balance due to the sensitivity of the eye [222,223]. Ensuring biocompatibility and maintaining normal eye function requires attention to factors like transparency, oxygen permeability, and mechanical integrity [224]. Oxygen permeability (Dk), which measures how well a lens allows oxygen to pass through, is a key property of the material [225]. Conversely, oxygen transmissibility (Dk/t) refers to the amount of oxygen that can penetrate a lens of a given thickness [226]. Poor oxygen transmissibility can result in eye hypoxia, discomfort, and acidosis of the epithelium, while higher Dk/t values are linked to better comfort and tolerance [167]. Hydrogels, a type of lens material, enhance oxygen permeability and offer several beneficial characteristics, such as supporting a stable tear film, allowing ion movement, and providing a non-irritating surface resistant to tear accumulation, formulating them biocompatible and comfortable [227].

Numerous hydrogel-based materials have been evaluated for use in ophthalmic drug delivery through soft contact lenses [228,229]. One promising option is PVA hydrogel, known for its elasticity, collagen-like mechanical features, and low friction coefficient, making it well-suited for eye care applications [230]. PVA-based contact lenses show high water content, translucence, and biocompatibility and are cost-effective. In vitro studies with human corneal epithelial cells (HCE-2s) demonstrated excellent cell viability, well above the ISO-defined cytocompatibility threshold, and appreciably better than the positive control (5% DMSO) [231].

**Figure 8:**
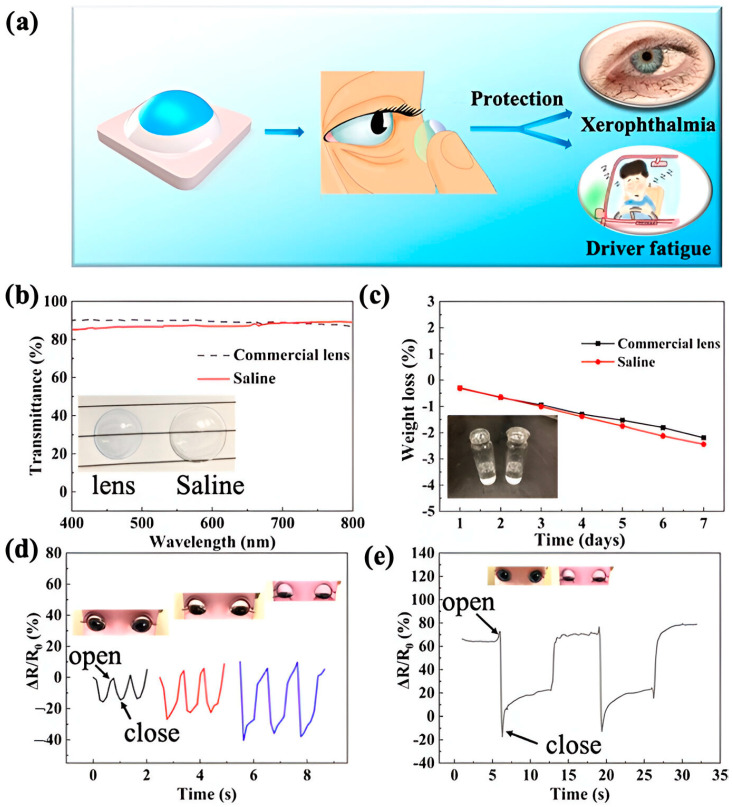
(**a**) Fabrication process and application of a PVA-based ionically conductive organohydrogel for smart contact lenses, treated with a saline solution. (**b**) Comparison of the transparency between a commercial contact lens and the PVA ionically conductive organohydrogel. (**c**) Measurement of weight loss over time at ambient temperature. (**d**) Variation in resistance ratio with different blinking frequencies. (**e**) Measurement of resistance change during prolonged eye closure on a body model. This figure is adopted with permission from the ref. [233]. Copyright © 2017, American Chemical Society.

Eye fatigue, characterized by a range of non-specific symptoms, is commonly associated with prolonged visual tasks and can lead to severe eye conditions if left unaddressed. For instance, computer vision syndrome, often linked to extended periods of computer use, and fatigue during driving, both present significant risks to public health and safety. Consequently, monitoring eye movements is crucial for detecting and preventing eye fatigue. The PVA-based ionic organohydrogel, known for its excellent linear sensitivity, high transparency, and biocompatibility, shows great promise as a material for smart contact lenses aimed at monitoring eye activity and supporting eye health [232]. In their study, Zha and colleagues introduce a co-solvent method for producing nanofibrillar PVA organohydrogels, which exhibit remarkable mechanical strength, clarity, sensitivity, and biocompatibility through a straightforward physical cross-linking process [233] (Figure 8).

Unlike conventional PVA hydrogels, which possess microscopic networks, these nanofibrillar organohydrogels, with a higher cross-linking density, display impressive tensile strength (1.4 MPa), toughness (~3.2 MJ m^–3^), and transparency (90%) [41]. By immersing the nanofibrillar PVA organohydrogel in a NaCl/glycerol/water solution, a transparent, biocompatible ionic organohydrogel is created. The network structure within these hydrogels supports stable, repeatable, and linear sensing capabilities, making them ideal candidates for applications in smart contact lenses and sensors that monitor both eye movements and human body signals. A prototype G1W1 organohydrogel lens (approximately 0.2 mm thick) was fabricated using a contact lens-shaped template and then treated with a saline solution to maintain osmotic balance with the eye environment [234]. This lens demonstrated comparable transmittance to commercial lenses, as shown in the accompanying figure.

Bacterial contamination is a major challenge in ocular therapy with contact lenses, reducing the therapeutic efficacy [235]. Kharagani et al. developed PVA hydrogel contact lenses incorporating silver and copper nanoparticles as antimicrobial agents [236]. The silver-loaded PVA hydrogel demonstrated strong antibacterial activity but was also highly toxic, while the copper-loaded hydrogel exhibited minimal toxicity. The antibacterial performance against Pseudomonas aeruginosa and Staphylococcus aureus was evident in inhibition zone assays, suggesting that these nanoparticle-infused hydrogels could effectively prevent infections and limit bacterial growth.

To enhance the therapeutic potential of contact lenses, several studies have explored PVA-based hydrogels as matrices for delivering bioactive compounds [237,238]. Various methods, including soaking the lenses in concentrated aqueous solutions of bioactive agents, can be used for this purpose. The soaking method involves the non-covalent adsorption of bioactive molecules onto the polymer matrix, followed by gradual release through molecular diffusion [239]. Factors such as solution concentration, water content, and polymer molecular weight play a significant role in determining the loading capacity and release kinetics [240,241]. While soaking is a simple and cost-effective approach, it does have limitations, including potential for burst release and lack of reproducibility. To address these challenges, Vitamin E is often used as a diffusion barrier. This antioxidant, known for its biocompatibility and therapeutic properties, helps to control drug release kinetics, ensuring sustained release while maintaining the functional integrity of the eye and the lens.

## 4. Conclusions and Future Perspectives

PVA-based materials offer significant advantages over existing technologies in terms of efficiency, cost, and scalability, making them stand out for therapeutic applications [242]. Their ability to form stable, biocompatible networks with excellent mechanical properties and responsiveness to external stimuli enhances their efficiency in drug delivery, wound dressings, and regenerative medicine. PVA is widely available and inexpensive, and its synthesis methods, such as physical gelation and chemical cross-linking, are straightforward and cost-effective, ensuring economic viability for large-scale production [243]. Additionally, PVA’s biocompatibility, non-toxic nature, and customizability allow it to be tailored for specific applications, such as combining with nanostructured materials to achieve advanced functionalities. Furthermore, PVA-based hydrogels are versatile and scalable, making them suitable for diverse applications, from biomedical uses to flexible electronics, while aligning with sustainability goals due to their water solubility and biodegradability. These features collectively position PVA-based materials as a competitive and often superior choice in the field.

PVA-based hydrogel possesses significant potential for biocompatibility and adjustable mechanical features, which are favorable in tackling the challenges encountered by conventional hydrogels and other polymers. Further, this type of polymer hydrogel can act as multifunctional materials that are appropriate for numerous applications in the biomedical and industrial sectors. Thus, the design and synthesis of PVA-based hydrogel utilize numerous fabrication techniques, including repeated freezing/thawing cycles in aqueous solutions, non-cryogenic physical gelation of PVA, chemical cross-linking of PVA, and so on, with better mechanical characteristics and responsiveness to external stimuli. Other potential properties may facilitate biomedical applications. Such unique features offer the pertinency of PVA-based hydrogels in numerous biomedical applications, such as tissue engineering and regenerative medicine, drug delivery, wound dressing, contact lenses, and cartilage substitution in joint and meniscal applications. Though considerable advancement occurred on PVA-based hydrogels, there are still possibilities to improve the biomedical applications for large-scale usages. In this context, mechanical strength, stability under different environmental atmospheres, and the toxicity of PVA-based hydrogels are vital concerns for their successful applications in biomedical sectors.

Additionally, the scalability of manufacturing procedures and the economic viability of such hydrogels should be addressed to enable their large-scale industrial applications. Therefore, more focus can be stipulated on the PVA-based hydrogels focusing on the easy and low-cost functionalization of them with their design and easy fabrication for biomedical applications. In future work, the research on PVA-based hydrogels can be focused on the incorporation of multi-functionalities including self-healing competences and superior multi-responsiveness to biological and chemical forms, the integration of nanocomposites, 3D printing approaches, and artificial intelligence. Such focus and inclusion may play a significant role in the preparation of smart-generation hydrogels, and the advancements will enhance the capabilities of PVA-based hydrogels as sustainable materials. Thus, owing to the flexibility of PVA-based hydrogels, more advancement in biomedical fields utilizing these materials is highly expected.

## Data Availability

No new data were created or analyzed in this study.
